# The Challenge of Producing Fiber-Based Organic Electronic Devices

**DOI:** 10.3390/ma7075254

**Published:** 2014-07-18

**Authors:** Tobias Könyves-Toth, Andrea Gassmann, Heinz von Seggern

**Affiliations:** Electronic Materials Division, Institute of Materials Science, Technische Universität Darmstadt, Alarich-Weiss-Str. 2, 64287 Darmstadt, Germany; E-Mails: koenyves@e-mat.tu-darmstadt.de (T.K.-T.); gassmann@e-mat.tu-darmstadt.de (A.G.)

**Keywords:** organic electronics, fibers, smart textiles, organic light emitting diodes, thin film deposition

## Abstract

The implementation of organic electronic devices on fibers is a challenging task, not yet investigated in detail. As was shown earlier, a direct transition from a flat device structure to a fiber substrate is in principle possible. However, a more detailed investigation of the process reveals additional complexities than just the transition in geometry. It will be shown, that the layer formation of evaporated materials behaves differently due to the multi-angled incidence on the fibers surface. In order to achieve homogenous layers the evaporation process has to be adapted. Additionally, the fiber geometry itself facilitates damaging of its surface due to mechanical impact and leads to a high surface roughness, thereby often hindering commercial fibers to be used as substrates. In this article, a treatment of commercial polymer-coated glass fibers will be demonstrated that allows for the fabrication of rather flexible organic light-emitting diodes (OLEDs) with cylindrical emission characteristics. Since OLEDs rely the most on a smooth substrate, fibers undergoing the proposed treatment are applicable for other organic electronic devices such as transistors and solar cells. Finally, the technique also supports the future fabrication of organic electronics not only in smart textiles and woven electronics but also in bent surfaces, which opens a wide range of applications.

## 1. Introduction

The vision of mass-produced smart textiles with electronic functionalities such as light emission or current switching for applications in textile displays, flexible lighting and wearable electronics is currently an interesting topic in textile research. In order to achieve this aim, a simple production and integration of semiconducting devices into textiles is in the focus of current research. Two different approaches are investigated presently, firstly, the integration of classical, inorganic electronic devices into fabrics [1] and, secondly, the development of new fiber-based device structures utilizing textile substrates [2]. Despite of their advantage of well-known electrical behavior and good performance, inorganic electronic devices have the disadvantage of being hard and rigid. Consequently, they oppose the main textile properties of being soft and flexible. In order to maintain these properties, the research of new OLED device structures assesses the possibility of integration without destroying the desired textile properties, however, ensuring economic production [1,2]. In this respect, organic electronics is an interesting alternative due to its thin layer structures (about 100 nm layer thickness) and their flexibility. Since most textiles consist of fabric woven or knitted from yarn, the possibilities of applying organic semiconductor devices directly onto fibers have been investigated. Reports from literature range from organic light-emitting diodes (OLEDs) [2,3] over organic field-effect transistors (OFETs) [4,5,6,7,8,9] to organic photovoltaic cells (OPV) [10,11,12,13,14]. However, only a few of the published devices cover the complete circumference of 360° of the fiber [2,3,10,11,12]. This becomes important if the size of the active area is of concern like in OLEDs or sensing applications. In principle, such fiber-based devices could be directly woven into the fabric and contacted in the same production step by conductive yarns [15,16], thereby opening the possibility of a potentially low cost production of textile-integrated electronics.

In their paper from 2007, O’Connor and coworkers [3] showed the working principle of a fiber-based small molecule OLED and compared it to a device in flat geometry. However, since that time nothing alike or new was reported for small molecule-based OLEDs on fibers. Consequently, the question arises what actually hampers further progress in fiber shaped small molecule-based organic devices. To answer this question a more detailed look on the processing of devices on fiber substrates is taken and will detail the processing-related challenges in this field. For these investigations it is mandatory to be able to reproducibly prepare rotationally symmetric devices. Thus, the first step was to develop a vacuum deposition system capable of meeting the necessary requirements for fiber substrates. In the custom-made system all process steps up to the complete device are executed automatically, starting from cleaned substrates. In the second step, the handling of the substrates during device preparation had to be elaborated and the layer formation was investigated separately. Finally, top emitting OLEDs were produced using a prototypical material stack [17] with process parameters adapted from flat geometry devices. In the course of these experiments critical parameters and material-related issues could be identified which will be discussed in detail. Especially the importance of the fiber substrate quality will be addressed and the question how to obtain smooth surfaces on commercially available fibers by a thermal treatment will be investigated.

## 2. Results and Discussion

### 2.1. Effect of the Transition from Flat to Cylindrical Substrate Geometry on Layer

**Figure 1:**
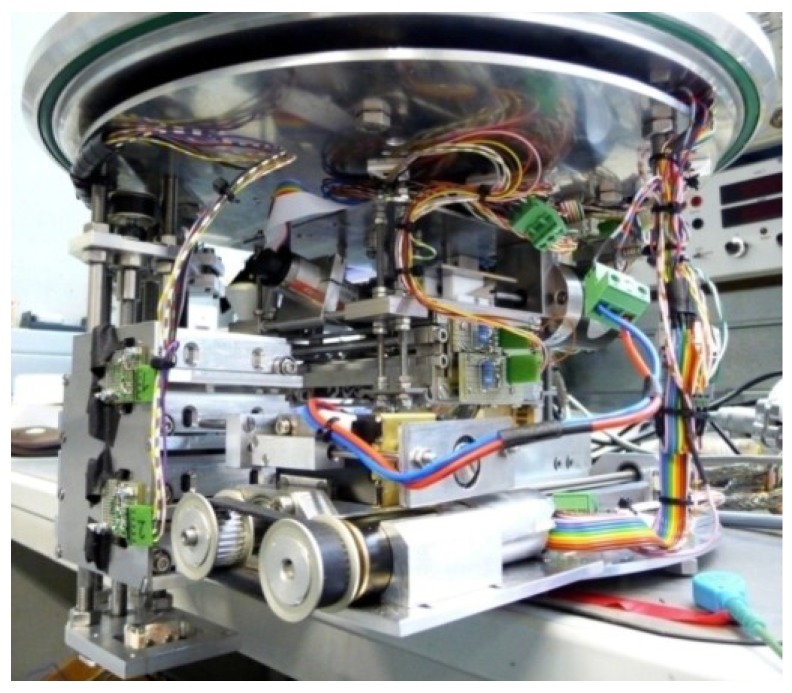
Photographic image of the developed automated system integrated into an evaporation chamber for the production of fiber-based organic electronic devices.

A reliable automated vacuum deposition system is essential for the investigation of a production process and its variation. In order to be able to fulfill all necessary requirements for fibers as substrates, a system was built from scratch. It was designed to fit into a vacuum deposition chamber (Auto 306 system from HHV Ltd., Crawley, UK) offering complete automation of device structuring by shadow masks and rotation of the fibers (see experimental section for a detailed description). Figure 1 shows a photographic image of the described system before its integration into the vacuum chamber.

For the investigation of possible necessary process alterations when changing from flat device structures to fibers, OLEDs instead of other devices like OFETs, *etc.*, were chosen as model devices. They exhibit a simple device structure and, additionally to electrical characterization, can also be evaluated optically. Furthermore, they have the highest requirements on the substrate surface quality making the results thus applicable to all other organic device structures. Due to their small layer thickness, organic semiconductor devices are in general very sensitive to the surface roughness of the substrate. To reliably fabricate functional OLEDs usually a root mean square (RMS) roughness below 1 nm is required [18]. In addition, for future textile applications mechanically flexible polymer fibers are mostly suited, however, problems in the surface quality have to be overcome. Therefore, initially rigid glass sticks with a diameter of 1 mm were used to allow for an easier substrate handling and to understand the influence of the cylindrical geometry on layer formation. Firstly, only the geometry was changed from a flat glass substrate to a cylindrical glass rod. To make sure that the glass surface of the substrates was not damaged, they were fabricated in house by glassblowers and stored without coming in contact to other surfaces.

It was found that basically all control parameters used for flat glass substrates, namely vacuum pressure, deposition rate and substrate temperature can be directly used for the deposition on rotated fibers. Only for calculation of the layer thickness, which is usually calibrated for a flat reference substrate, the ratio of circumference to diameter π has to be taken into account. In addition, it was discovered that the rotation speed of the fiber substrates is an additional control parameter, which has to be adjusted.

**Figure 2:**
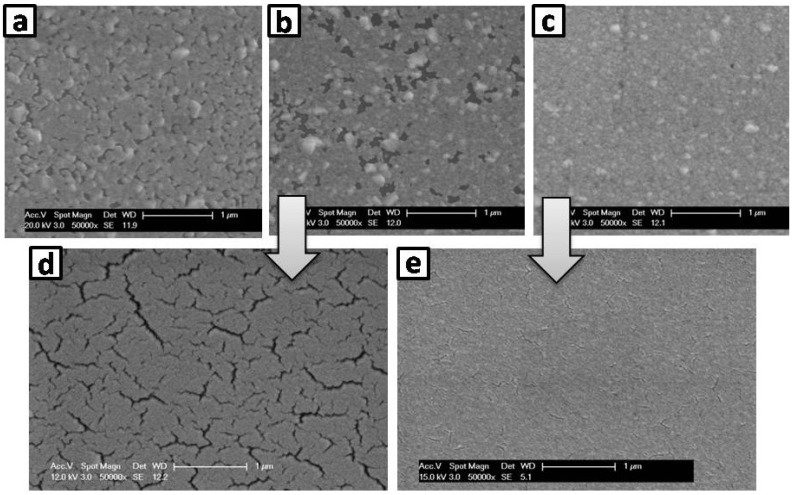
Scanning electron microscope images of Ag and Au surfaces. All layers were deposited with varying rotation speeds onto glass sticks (diameter 1 mm) coated with 110 nm PEDOT:PSS. (**a**) 20 nm Ag (no rotation); (**b**) 20 nm Ag (4 rpm); (**c**) 20 nm Ag (0.5 rpm); (**d**) 30 nm Au (4 rpm) on 20 nm Ag (4 rpm); (**e**) 30 nm Au (4 rpm) on 20 nm Ag (0.5 rpm).

It is known, that the layer growth and morphology of thin films depends for oblique evaporation on the incident angle [19]. By changing the angle of incidence during layer deposition it is thus possible to change the properties of the layer. For a fiber geometry, multiple angles are present at the same time. This results in an even more complex layer growth, which was found to be directly affected by the rotation speed of the fibers. In addition experiments require a poly(3,4-ethylenedioxythiophene) poly(styrene-sulfonate) (PEDOT:PSS) coating of the substrates as is explained below. It acts as adhesive and nucleation layer for the silver layer used as underlying layer for the anode [20]. Figure 2 shows the morphology of silver layers deposited at different rotation speeds on PEDOT:PSS-coated glass sticks. The layers produced on non-rotated fibers (Figure 2a) and on at a rotation speed of 4 rpm rotated fibers (Figure 2b) show an incomplete surface coverage, leading to cracks in the on top evaporated gold layer that was used as anode in the OLEDs (Figure 2d). These cracks resulted in a fast burn-out of the respective OLEDs. However, using a fiber rotation speed of 0.5 rpm a complete coverage of the PEDOT:PSS-coated glass stick by the silver layer could be achieved (Figure 2c), leading to a considerable reduction in the number and size of cracks in the Au layer and to more stable OLED devices (see Figure 2e). O’Connor *et al.* [3] utilized 30 rpm on a polyimide coated fiber and thus demonstrated that faster rotation can also yield good results. Although we do not yet understand the mechanism why, in our case, a slow fiber rotation is superior to a faster one, the adhesion of the metal on the glass surface plays an important role as stated above. Additionally silver layers without an underlying PEDOT:PSS layer always showed an incomplete coverage (comparable to Figure 2b) independent of the used rotation speed. Consequently devices without PEDOT:PSS were not functional due to immediate burn-out like the devices with polymer coating and a rotation speed of 4 rpm.

The layer formation of the first metallic layers deposited on the substrate is crucial for the functionality of the final OLED due to the above-mentioned requirements. In contrast to the relevance of the layer quality of the polycrystalline metallic layers, the layer formation of the organic small molecules is not an issue. The prototypical organic semiconductors used are known to form amorphous layers with a smooth surface [21,22].

### 2.2. Electrical and Optical Device Properties

**Figure 3:**
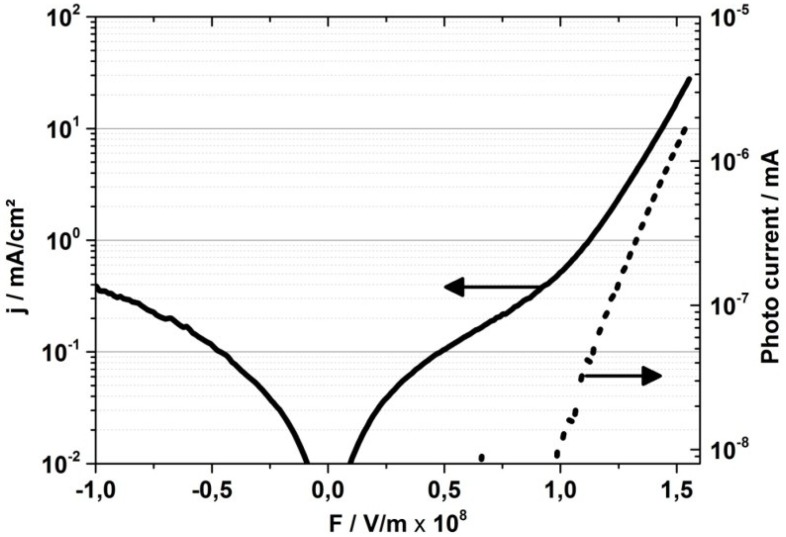
Electrical characteristics of a 1 mm long OLED processed on a defect-free glass stick (diameter 1 mm).

**Figure 4:**
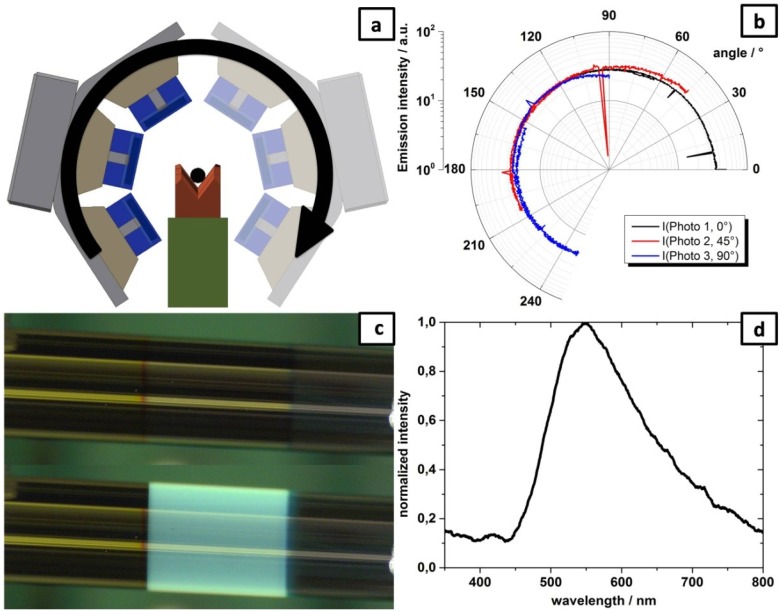
(**a**) Schematic of the measurements setup for angle dependant emission. The fiber (black, **center**) is contacted by laying in contact notches and the ensemble of photodiodes (blue) moved on a fixed radius around the OLED; (**b**) Angle dependence of light emission of an OLED processed on a defect-free glass stick using three photodiodes that were rotated around the device; (**c**) Photographs of the 1 mm OLED in the off (**top**) and on state (**bottom**); (**d**) Electroluminescence spectra of the diode.

After having evaluated the effect of the substrate rotation speed on the formation of the metallic layers used as anode, small molecule OLEDs were prepared and electro-optically characterized (see Section 3 for experimental and device structure details). Figure 3 shows the current density *j* as a function of the electric field *F* of an OLED on a glass stick (1 mm diameter) with 1 mm length, which was measured in dry nitrogen atmosphere using a HP 4155A semiconductor parameter analyzer and a photodiode (BPW 34, Osram, Regensburg, Germany). It has to be noted that the electric field corresponds to voltages of up to 21 V and is thus higher than typically applied to flat OLEDs using the same organic semiconductors [17]. This mainly results from the voltage drop along the cathode which on the one hand has to be kept thin to out-couple the generated light (see Figure 4) but resulting on the other hand in a high resistivity. This influence can be reduced if the device is contacted directly in the region of the active area, however this was avoided to minimize the risk of damaging the layers by the contact needles.

The angle dependence of the light emission was also measured using an ensemble of three photodiodes (Siemens BPW 34B, Munich, Germany). They were moved at a fixed radius and at an angle of 45° to each other around the OLED, as is depicted in Figure 4a. Consequently the diodes are covering a total angle of up to 250° around the circumference and verifying each other’s results by the overlapping range. A coverage of the whole 360° circumference was not possible due to the mounting and contacting of the fiber. The result is plotted in Figure 4b and shows a homogenous emission in all directions. The measured higher emission intensities at low angles (below 90°) are related to a slightly non-centric positioning of the OLED in the setup and do not represent a real local increase in emission intensity. The sudden decrease in the signal of photodiode 2 at about 95° is attributed to a loss of contact at this angle. Since the photodiodes 1 and 3 stay at level, they verify the result. The same can be seen in the signal of photodiode 1 between 0° and 60°. In Figure 4 the photographs of the measured OLED in the on and off state are given. From the bottom picture of Figure 4c one can clearly see that the cathode stack is semitransparent. At the electroluminescence maximum (530 nm for Alq_3_), the transmission loss is about 50% for the used cathode layer thickness. Even though the photograph of the switched on OLED seems to emit rather bluish, the measured electroluminescence spectrum in Figure 4d proves that the emission maximum is indeed in the green wavelength range as expected from Alq_3_. The seemingly observed color change is actually an artifact of the camera, due to a non-adjusted white balance. Nevertheless, the photo nicely illustrates the homogeneous light emission of the device.

### 2.3. Smooth Surfaces on Commercial Fibers

Even though the well-performing OLEDs on defect-free glass sticks proved that these substrates are in general suitable for organic devices, the approach is not applicable for smart textiles. The reason is that a sufficient surface quality is in general not given for commercial fibers as will be detailed below. Thus, the obtained processing technology for cylindrical substrates has to be implemented for mechanically flexible, commercially available optical fibers. Thereby, the successful preparation of fiber-based devices shifts back to the question of the surface quality of the fiber itself and to means to smoothen it.

In general, the common way to get smooth (flat) substrates is the fabrication of the surface from molten materials, since surface tension tries to minimize the surface energy. Like the common float glass, these substrates have a very low surface roughness. Basically, the same holds true for fibers spun from the melt. However, contrary to flat glass substrates, the smooth surface is not easily maintained in the case of fibers. The storage of fibers (and yarns) usually includes a contact of the fiber surface to other objects, leading to a damage of the surface by mechanical scratches. The obvious example is the yarn on a bobbin scratching itself while being coiled up. The same holds true for the transport of the yarn by guiding systems in the production process. To protect the optical fiber from such damages a buffer coating or cladding is usually applied. In the case of glass fibers this coating also provides the mechanical integrity of the whole fiber.

Since, in general, no fresh and, thus, undamaged optical fibers are available, a re-melting of the surface could heal failures. However, due to their low ratio of volume to surface a sole melting of the surface is difficult to achieve. Thus, unclad fibers will lose their form and/or diameter when heated above their melting temperature. In this respect, glass fibers coated with a buffer layer may have an advantage. If treated properly the surface of a polymeric buffer layer can be melted while the actual glass fiber does not lose its cross sectional shape and its diameter due to its high melting point which keeps the surface of the cladding in its form.

**Figure 5:**
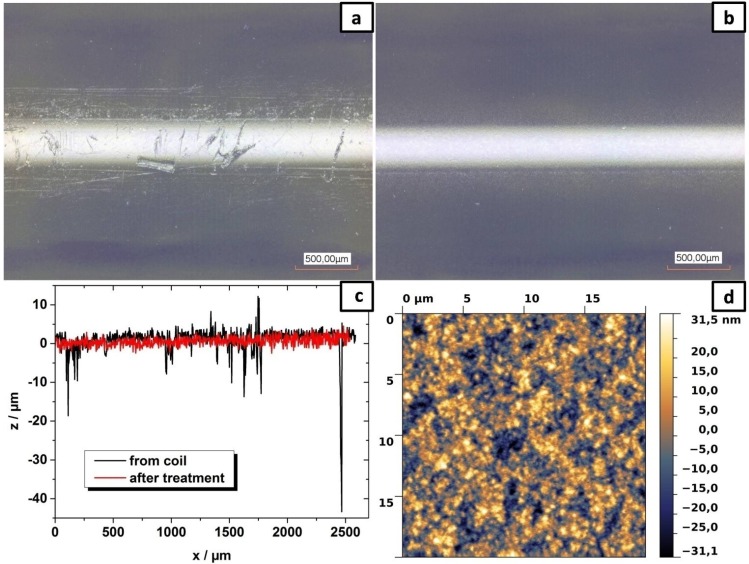
(**a**) 3D laser scanning microscope images of the same ETFE-coated optical fiber before (from coil) and (**b**) after the temperature treatment; (**c**) The surface profile shows that the peak-to-peak roughness is reduced from about 50 μm to a value lower than the resolution of the imaging method; (**d**) AFM image of the ETFE surface after treatment. The resulting RMS surface roughness is 11 nm.

To prove the above-described principle of a temperature treatment hard-clad silica (HCS) optical fibers encased in a buffer layer of ethylene tetrafluoroethylene (ETFE) with two different diameters were utilized. The thicker fibers with an outer diameter of 1040 μm had a HCS core diameter of 630 μm and a buffer layer thickness of 205 μm. The thinner fibers with an outer diameter of 500 μm exhibited a HCS core of 230 μm in diameter and the ETFE buffer was 135 μm thick. For the investigation of the as received fiber surface and the ETFE buffer roughness, a 3D laser scanning microscope (Confocal Laser Scanning Microscope VK9700, KEYENCE, Neu-Isenburg, Germany) was used allowing for the recording of the radial surface roughness. The image of the surface of the 1040 μm thick, commercial HCS fiber is depicted in Figure 5a and shows various deep scratches. The peak-to-peak roughness reaches values of about 42 μm, making this fiber inapplicable as OLED substrate. The melting temperature of ETFE was measured by differential scanning calorimetry (DSC 200 F3 Maia^®^, Netzsch Gerätebau GmbH, Selb, Germany) to be 250 °C in agreement with literature [23]. A temperature treatment of the fibers in an oven at a temperature exceeding the melting point of ETFE results in the desired smooth surface (see Section 3 for experimental details). In Figure 5, the image of the fiber surface and its height profile after thermal treatment are depicted in comparison to the original state. It should be noted that the same part of the fiber is shown. The resulting height profile displays a roughness below the resolution limit of the laser scanning microscope. The investigation of the surface by atomic force microscopy (AFM) is included in Figure 5 and yields an RMS surface roughness of 11 nm, which is consistent with published values for ETFE [24]. Even though the roughness of the surface is still higher than recommended for OLEDs (r(RMS) < 1 nm), these substrates can already be successfully used as will be demonstrated in the following. In principle the described smoothing process should be applicable to every material combination as long as the core of the fiber has a sufficiently higher melting point than the mantle material.

### 2.4. OLEDs on Thermally Smoothened HCS Fibers

**Figure 6:**
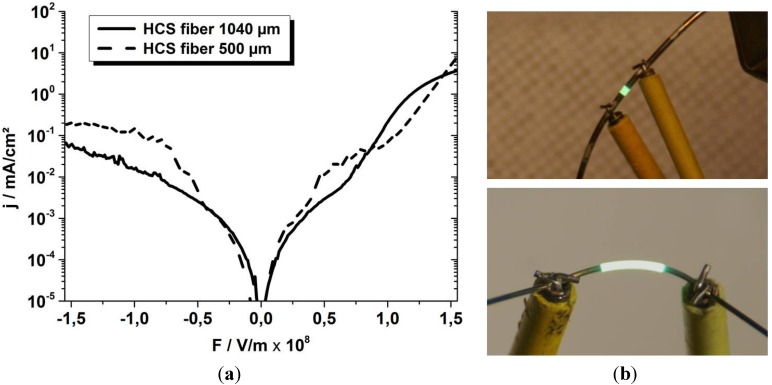
(**a**) *j*-*F*-characteristics and (**b**) photographs of a 1 mm OLED produced on a 1040 μm thick ETFE-coated, thermally smoothed HCS fiber (**top**) and a 5 mm OLED produced on a 500 μm thick HCS fiber treated the same way (**bottom**).

The *j*-*F*-characteristics of OLEDs processed on thermally smoothened ETFE-clad optical fibers with different diameters are shown in Figure 6. One OLED is 1 mm long and has been processed on a 1040 μm thick fiber, thus having an active area of 3.3 mm^2^. The other OLED has been prepared on a thinner fiber of 500 μm diameter, using a larger device length of 5 mm yielding an active area of 7.9 mm^2^. For measuring the light emission the used photo diodes were not sensitive enough. The photographs shown in Figure 6 showing the OLEDs in on state were taken at exposure times of up to 120 s to capture the working diode. As can be deduced from Figure 6b the fibers are already quite flexible and even for bend devices the OLEDs are still functioning. However, the *j*-*F*-characteristics of the devices processed on the thinner fibers show some artifacts in the range from 40 MV/m to 90 MV/m resembling a negative differential resistance, which could result from the high surface roughness of 11 nm of the thermally treated ETFE and therewith of the electrode interfaces [25]. Furthermore, the devices are not yet stable to withstand repeated or alternated bending, yielding currently an electrical device failure by a short circuit after 10 bending cycles to a radius of 10 mm. The reason for the failure is yet to be determined. Due to the failure by electric short circuit we assume it to be related to a crack propagation of the still existing cracks in the anode (compare Figure 2e) leading to a higher charge injection at the cracks and thus burn out of the devices.

### 2.5. Other Routes to Improve the Surface Quality of All-Polymeric Fibers

To even further reduce the surface roughness other smoothing techniques were investigated. In the production of OLEDs a remaining surface roughness is usually treated by the application of a second layer in order to smoothen it. A prominent example for this technique is the PEDOT:PSS-coating of transparent conductive oxides like indium tin oxide (ITO) [18]. A prerequisite for this technique is that the employed solution or dispersion of the material to be coated shows a good wetting of the substrate surface. This is, unfortunately, not the case for the above discussed ETFE-clad glass fibers due to the high surface tension [24]. Consequently, all polymeric optical fibers were chosen in order to exploit other smoothing strategies, even though they were unsuited as OLED substrates. Due to the lack of the high melting core the thermal smoothing of the scratched surface was not applicable for these fibers and the surface consequently too rough to support the thin device structures.

Investigating as received, unclad polymeric fibers the areas between the deep scratches showed actually a surface roughness in the range of the heat-treated ETFE cladding on HCS fibers. In these regions it is legitimate to assume that comparable devices to those of the heat-treated glass fibers with ETFE-cladding can be obtained. In order to improve the surface roughness additional coatings can be applied as mentioned above. In the case of poly(methyl methacrylate) (PMMA) fibers it could be demonstrated that an additional coating with two different polymer layers, consisting of polyvinyl alcohol (PVA) and PMMA reduced the surface roughness below 1 nm. During the processing the use of orthogonal solvents is mandatory in order to not dissolve the fiber itself, nor the first coated polymer layer. To smoothen the PMMA fibers (diameter 500 μm) both layers were applied from solution using dip coating (see Section 3 for experimental details).

**Figure 7:**
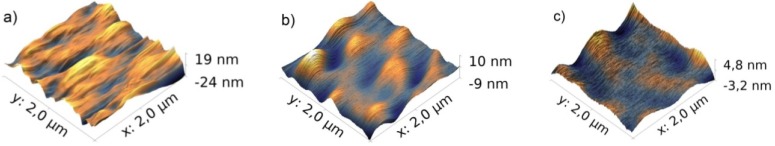
The surface of a PMMA fiber as imaged by AFM. The corresponding RMS roughness is given in brackets: (**a**) Untreated fiber (5 nm); (**b**) fiber after coating with PVA (2.4 nm); (**c**) fiber after subsequent coating with PVA and PMMA (0.9 nm).

The fibers showed an initial RMS surface roughness of 5 nm as imaged by AFM (Figure 7a). Applying the PVA layer results in a reduction of the roughness to 2.4 nm, about half of the initial value (Figure 7b). After the second, subsequently applied PMMA coating the final roughness was measured to be 0.9 nm (Figure 7c) and, thus, meets the initially discussed requirements for OLED applications. These results suggest that a fiber with a high melting core encased in a mantle of PMMA should be perfectly suitable for OLEDs after the described thermal smoothing and subsequent coating with polymer solutions.

These findings can be utilized to further speed-up the development and application of fiber-based devices. Since methods to control the fiber substrate surface quality and the importance of the adjustment of the rotation speed during the layer deposition have been demonstrated, the focus of future experiments can be directed to the development of concepts for a continuous manufacturing process and to exploit methods to contact functional devices on fibers by conductive yarns. There is still a long way to go to the integration of organic semiconductor-based devices into textile applications, yet first necessary steps have been demonstrated.

## 3. Experimental Section

Prior to the deposition of the active layers all fiber substrates were cleaned by a sequence of washing steps in an ultrasonic bath. First, the substrates were rinsed in deionized water (DI-water) and subsequently washed in a solution of the detergent Deconex (Deconex PF15, 5 wt% in DI-water, Borer Chemie AG, Zuchwil, Switzerland) at 60 °C for 15 min. In the second step, they were washed in DI-water at room temperature for 15 min to remove all excess Deconex solution left on the fibers. In the last step the fibers were washed in 2-propanol (VLSI Selectipur^®^, BASF, Rhine, Germany) at room temperature for 15 min. After drying the substrates in a nitrogen stream they were ready to be coated with the adhesion promoting layer of PEDOT:PSS (Clevios AI4083 aqueous dispersion, Heraeus, Hanau, Germany). This is done by dipping the fibers into the PEDOT:PSS-dispersion and withdrawing them at a speed of 80 mm/min, resulting in a layer thickness of 110 nm. Afterwards, the substrates are mounted in the sample holder to be transferred into the deposition chamber.

In the vacuum deposition system the sample holder for the fiber substrates can be decoupled from the fiber’s rotation drive to allow the sample transfer via a load lock in inert nitrogen atmosphere. This is important especially after the process, since the small molecule-based organic semiconductors are in general sensitive to moisture and oxygen. The sample holder can hold up to 5 fibers with a length of 70 mm each, of which 50 mm can be structured. Mounted fibers can be rotated centric and homogeneously at speeds of up to 5 rpm. For the structuring of the OLED layers the system can *in situ* access 4 shadow masks. These masks are mounted in a magazine and can be positioned under the rotating fibers, if required. Monitoring quartz sensors allow the system to be controlled via a computer interface. To fully utilize the capability of the system the evaporation sources of the vacuum chamber were modified to hold up to 8 resistively heated crucibles. The active material source can be switched *in situ* without venting the vacuum chamber, enabling the system to hold the base pressure below 10^−6^ mbar during the whole evaporation process. The system is completed by a home-made transfer system that can be attached to the chamber thereby allowing for a sample transfer to the glove box where the electrical measurements are performed.

**Figure 8:**
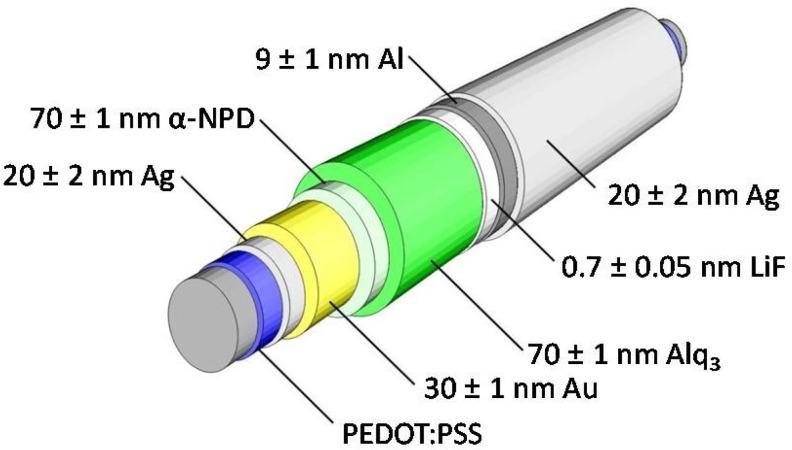
Illustration of the fiber OLED layer sequence. All materials were deposited by subsequent resistive evaporation in a vacuum chamber.

Since gold is easily scratched off the glass surface, a better adhesion and contacting of the Au anode was achieved by depositing an underlying layer of 20 ± 2 nm Ag before 30 ± 1 nm Au were deposited. The anode was followed by a double layer of organic semiconductors. *N*,*N*′-Di-[(1-naphthyl)-*N*,*N*′-diphenyl]-1,1′-biphenyl)-4,4′-diamine (α-NPD) served as hole transport layer and tris-(8-hydroxyquinoline) aluminum (Alq_3_) was used as emitter and electron transport layer. Both layers had a thickness of 70 ± 1 nm, respectively. Finally, 0.7 ± 0.05 nm of LiF, 9 ± 1 nm of Al and 20 ± 2 nm of Ag were deposited as a semitransparent cathode stack. All materials were deposited by resistive evaporation in a vacuum chamber utilizing deposition rates of 1–2 Ås^−1^ (0.1 Ås^−1^ for LiF) and pressures of 5 × 10^−7^ to 3 × 10^−6^ mbar. The deposition was only stopped at full rotations in order to get a homogeneous layer thickness over the whole circumference. Thus variations of the deposition rate resulted in the above-mentioned variations in the layer thickness since the deposition was stopped e.g., one rotation earlier or later. With the exception of the Ag layer, all other layers were deposited at a rotation speed of 4 rpm. For the Ag layers underlying the anode and the layer covering the semitransparent cathode 0.5 rpm and 2 rpm were used, respectively. Per substrate, 3 to 5 OLED devices were structured using shadow masks. The overlapping electrodes resulted in OLEDs of 1 mm or 5 mm length. The substrates where not heated. Over the whole process their temperature never exceeded 60 °C. Figure 8 illustrates the described device structure.

For the smoothing of the ETFE-clad HCS fibers, they were cleaned properly prior to annealing in order to avoid any partial embedment of, e.g., dust particles in the molten buffer or the creation of holes in the surface during the exposure to high temperatures. The same cleaning as described above was used. Afterward the fibers were annealed at 280 °C for 15 min. and after removing from the oven cooled to room temperature. The following evaporation of the OLED was executed without additional cleaning.

For investigation of the polymer smoothing the PMMA fiber was cleaned as above and subsequently coated by dip coating. In order to not dissolve the PMMA fiber surface, an aqueous solution of polyvinyl alcohol (PVA from Sigma Aldrich, Steinheim, Germany; 3 wt% in DI-water) was dip coated first and the fiber was subsequently dried for 5 min at 105 °C in an oven. In the second step the fiber was coated using a PMMA-solution (PMMA from Sigma Aldrich, Steinheim, Germany; 6 wt% in toluene). Again the fiber was dried for 5 min at 105 °C in an oven. Both coatings where applied using a dip coating speed of 80 mm/min.

## 4. Conclusions

Even though a number of organic semiconductor-based devices have been introduced a couple of years ago a reliable and simple production of these devices is still challenging. In the current work substrate- and processing-related questions have been investigated in more detail in order to identify the most critical parameters in the production process. The devices under investigation are OLEDs due to their high requirements on the substrate quality, *i.e.*, the surface roughness. To investigate OLED devices on cylindrical substrates, especially on fibers, the first step of the present work was the development and integration of an evaporation system capable of rotating and structuring fibers in a vacuum chamber. It has been found that the transition from flat to cylindrical substrate geometry can be achieved only, if a homogenous layer formation is ensured by the proper adjustment of the substrate rotation speed. However, the precondition for functional devices is still a defect-free (scratches, corrugations, holes...) surface of the substrate itself. This can be ensured by either a fresh preparation of the fiber substrates or by a proper surface treatment of commercial optical fibers that are usually damaged due to production- or storage-related issues. A thermal treatment of polymer-mantled fibers with a high melting core has been demonstrated to reduce the surface roughness to a level that allows for the successful production of functional OLEDs with cylindrical emission characteristics. Due to a high melting point core the whole fiber stays in shape during the thermal treatment. Only the mantle material is melted and smoothened thereby removing deep scratches. Additional polymeric layers applied by dip coating of suited materials from orthogonal solvents can reduce the surface roughness further to below 1 nm thereby enabling an improved device performance of the OLEDs. In summary, the present work has identified the important parameters essential for the reliable and reproducible production of fiber-based organic devices thus further paving the way to future applications like in smart textiles.

## References

[1] Cherenack K., van Pieterson L. (2012). Smart textiles: Challenges and opportunities. J. Appl. Phys..

[2] Janietz S., Gruber B., Schattauer S., Schulze K. (2013). Integration of OLEDs in Textiles. Adv. Sci. Technol..

[3] O’Connor B., An K.H., Zhao Y., Pipe K.P., Shtein M. (2007). Fiber shaped light emitting device. Adv. Mater..

[4] Lee J.B., Subramanian V. Organic transistors on fiber: A first step towards electronic textiles. Proceedings of the IEEE International Electron Devices Meeting, 2003 (IEDM’03 Technical Digest).

[5] Lee J.B., Subramanian V. (2005). Weave patterned organic transistors on fiber for E-textiles. IEEE Trans. Electron Devices.

[6] Bonfiglio A., de Rossi D., Kirstein T., Locher I.R., Mameli F., Paradiso R., Vozzi G. (2005). Organic field effect transistors for textile applications. IEEE Trans. Inf. Technol. Biomed..

[7] Maccioni M., Orgiu E., Cosseddu P., Locci S., Bonfiglio A. (2006). Towards the textile transistor: Assembly and characterization of an organic field effect transistor with a cylindrical geometry. Appl. Phys. Lett..

[8] Cosseddu P., Mattana G., Orgiu E., Bonfiglio A. (2009). Ambipolar organic field-effect transistors on unconventional substrates. Appl. Phys. A.

[9] Jang J., Nam S., Hwang J., Park J.J., Im J., Park C.E., Kim J.M. (2012). Photocurable polymer gate dielectrics for cylindrical organic field-effect transistors with high bending stability. J. Mater. Chem..

[10] Toivola M., Ferenets M., Lund P., Harlin A. (2009). Photovoltaic fiber. Thin Solid Films.

[11] Lee M.R., Eckert R.D., Forberich K., Dennler G., Brabec C.J., Gaudiana R.A. (2009). Solar power wires based on organic photovoltaic materials. Science.

[12] Liu J., Namboothiry M.A., Carroll D.L. (2007). Fiber-based architectures for organic photovoltaics. Appl. Phys. Lett..

[13] Bedeloglu A.C., Demir A., Bozkurt Y., Sariciftci N.S. (2010). A photovoltaic fiber design for smart textiles. Text. Res. J..

[14] O’Connor B., Pipe K.P., Shtein M. (2008). Fiber based organic photovoltaic devices. Appl. Phys. Lett..

[15] Hamedi M., Forchheimer R., Inganäs O. (2007). Towards woven logic from organic electronic fibres. Nat. Mater..

[16] Cherenack K., Zysset C., Kinkeldei T., Münzenrieder N., Tröster G. (2010). Woven electronic fibers with sensing and display functions for smart textiles. Adv. Mater..

[17] Gassmann A., Melzer C., von Seggern H. (2009). The Li_3_PO_4_/Al bilayer: An efficient cathode for organic light emitting devices. J. Appl. Phys..

[18] Jonda C., Mayer A.B.R., Stolz U., Elschner A., Karbach A. (2000). Surface roughness effects and their influence on the degradation of organic light emitting devices. J. Mater. Sci..

[19] Taschuk M.T., Hawkeye M.M., Brett M.J., Martin P.M. (2010). Glancing angle deposition. Handbook of Deposition Technologies for Films and Coatings.

[20] Ke L., Lai S.C., Liu H., Peh C.K.N., Wang B., Teng J.H. (2012). Ultrasmooth silver thin film on PEDOT:PSS nucleation layer for extended surface plasmon propagation. ACS Appl. Mater. Interfaces.

[21] Farahzadi A., Niyamakom P., Beigmohamadi M., Meyer N., Keiper D., Heuken M., Ghasemi F., Rahimi Tabar M.R., Michely T., Wuttig M. (2010). Stochastic analysis on temperature-dependent roughening of amorphous organic films. Europhys. Lett..

[22] Zhang F., Xu Z., Zhao D., Zhao S., Jiang W., Yuan G., Song D., Wang Y., Xu X. (2007). Influence of evaporation conditions of Alq_3_ on the performance of organic light emitting diodes. J. Phys. D Appl. Phys..

[23] Liu Z., Song Y., Shangguan Y., Zheng Q. (2007). Conductive behavior of composites composed of carbon black-filled ethylene-tetrafluoroethylene copolymer. J. Mater. Sci..

[24] Lee S., Park J.S., Lee T.R. (2008). The wettability of fluoropolymer surfaces: Influence of surface dipoles. Langmuir.

[25] Kolesnikov V.A., Zolotarevsky V.I., Vannikov A.V. (2003). Anomalous current-voltage characteristics of thin polymer films. Phys. Status Solidi A.

